# Self-Care for Common Colds by Primary Care Patients: A European Multicenter Survey on the Prevalence and Patterns of Practices—The COCO Study

**DOI:** 10.1155/2016/6949202

**Published:** 2016-09-21

**Authors:** Anika Thielmann, Biljana Gerasimovska-Kitanovska, Krzysztof Buczkowski, Tuomas H. Koskela, Vildan Mevsim, Slawomir Czachowski, Ferdinando Petrazzuoli, Marija Petek-Šter, Heidrun Lingner, Robert D. Hoffman, Selda Tekiner, Juliette Chambe, Tamer Edirne, Kathryn Hoffmann, Enzo Pirrotta, Ayşegül Uludağ, Hülya Yikilkan, Sanda Kreitmayer Pestic, Andrzej Zielinski, Clara Guede Fernández, Birgitta Weltermann

**Affiliations:** ^1^Institute for General Medicine, University Hospital Essen, University of Duisburg-Essen, Hufelandstr. 55, 45147 Essen, Germany; ^2^Department of Family Medicine and Department of Nephrology, University of St. Cyril and Methodius, Str. Vodnjanska 17, Skopje, Macedonia; ^3^Department of Family Medicine, Nicolaus Copernicus University, Sklodowskiej-Curie 9, 85-094 Bydgoszcz, Poland; ^4^Department of General Practice, University of Tampere, Lääkärinkatu 1, 33014 Tampere, Finland; ^5^Department of Family Medicine, Dokuz Eylul University Faculty of Medicine, Izmir, Turkey; ^6^Collegium Medicum, Nicolaus Copernicus University, Grabowa 10, 87-100 Toruń, Poland; ^7^Department of Clinical Sciences in Malmö, Centre for Primary Health Care Research, Lund University, Malmö, Sweden; ^8^Department of Family Medicine, University of Ljubljana, Poljanski Nasip 58, Sl-1000 Ljubljana, Slovenia; ^9^Centre for Public Healthcare, Hannover Medical School, Carl-Neuberg-Straße 1, 30625 Hannover, Germany; ^10^Departments of Family Medicine and Medical Education, Saklar Medical School, Tel Aviv University, 8 Gordon Street, 76291 Rehovot, Israel; ^11^Ankara University School of Medicine, Ibn-i-Sina Hospital Department of Family Medicine, Samanpazari, 06100 Ankara, Turkey; ^12^Department of General Practice, University of Strasbourg, 2a rue de Brantôme, 67100 Strasbourg, France; ^13^Department of Family Medicine, University of Pamukkale, PAU Tip Fakultesi, Aile Hekimligi AD, Kinikli Kampus, Denizli, Turkey; ^14^Department of General Practice and Family Medicine, Center for Public Health, Medical University of Vienna, Kinderspitalgasse 15, 1st Floor, 1090 Vienna, Austria; ^15^SNAMID, Italian Society of General Practitioners, ASL Roma B, Via Tuscolana, 859 Rome, Italy; ^16^Faculty of Medicine, Department of Family Medicine, Çanakkale Onsekiz Mart University, Terzioğlu Campus, 17100 Çanakkale, Turkey; ^17^Family Medicine Department, Diskapi Yildirim Beyazit Training and Research Hospital, Irfan Basbug Cad., Diskapi, 06110 Ankara, Turkey; ^18^Medical Faculty Tuzla, Family Medicine Department, Health Center Tuzla, Department for General/Family Medicine, Marsala Tita 199, 75000 Tuzla, Bosnia and Herzegovina; ^19^Blekinge Centre of Competence, Lyckeby Primary Health Care Centre, Källevägen 12, 37162 Lyckeby, Sweden; ^20^EOXI Vigo, University of Vigo, Calle Zaragoza, Vigo, 36203 Pontevedra, Spain

## Abstract

*Background.* Patients use self-care to relieve symptoms of common colds, yet little is known about the prevalence and patterns across Europe.* Methods/Design.* In a cross-sectional study 27 primary care practices from 14 countries distributed 120 questionnaires to consecutive patients (≥18 years, any reason for consultation). A 27-item questionnaire asked for patients' self-care for their last common cold.* Results.* 3,074 patients from 27 European sites participated. Their mean age was 46.7 years, and 62.5% were females. 99% of the participants used ≥1 self-care practice. In total, 527 different practices were reported; the age-standardized mean was 11.5 (±SD 6.0) per participant. The most frequent self-care categories were foodstuffs (95%), extras at home (81%), preparations for intestinal absorption (81%), and intranasal applications (53%). Patterns were similar across all sites, while the number of practices varied between and within countries. The most frequent single practices were water (43%), honey (42%), paracetamol (38%), oranges/orange juice (38%), and staying in bed (38%). Participants used 9 times more nonpharmaceutical items than pharmaceutical items. The majority (69%) combined self-care with and without proof of evidence, while ≤1% used only evidence-based items.* Discussion.* This first cross-national study on self-care for common colds showed a similar pattern across sites but quantitative differences.

## 1. Introduction

Common colds are the most frequently encountered human diseases worldwide [1]. Common cold is a conventional term used for mild upper respiratory illnesses, which comprises a heterogeneous group of self-limited diseases caused by numerous viruses. The frequency is age-specific with fewer episodes in adults than in younger children [2, 3] and a seasonal variation with more episodes in winter and fall [1]. Beyond impairing the quality of life [4], common colds have a tremendous economic burden on societies due to work absenteeism (70 million workdays annually due to non-influenza-related viral respiratory tract infections in the US, which corresponds to US$8 billion indirect costs) [5–7]. If consulted, general practitioners are asked to provide supportive advice, symptomatic treatment, and reassurance [8].

Studies on self-care have shown that common colds are the most frequent cause for self-care [9–12]. This is reasonable, as common colds have a self-limited course and resolve without treatment. Unsystematic practice observations and the few studies available indicate that patients use a mixture of evidence-based and non-evidence-based self-care practices including self-medication and traditional home remedies to reduce symptoms and improve subjective well-being (e.g., [9, 12–14]). Nevertheless, only few self-care practices have been studied and proven to be effective in relieving symptoms of common colds in adults, such as nonsteroidal anti-inflammatory agents [15], antihistamines [16], and oral antihistamine-decongestant-analgesic combinations [17].

Although self-care for minor illnesses is recognized as an important component of primary health care, little is known about the prevalence and patterns of self-care for common colds across Europe. Drawing on data from 27 primary care sites of 14 countries, the cross-sectional COCO study describes the variety and pattern of reported self-care practices.

## 2. Methods and Materials

### 2.1. Study Design

This multicenter, cross-sectional survey was performed at 27 primary sites in 14 European countries. Details of the study design have been published earlier [18]. Briefly, the cross-national survey was designed and conducted by the Working Group on Self-Care of the European General Practice Research Network (EGPRN). The data collection was completed in April, 2014.

### 2.2. Study Instrument and Data Collection

A 27-item self-administered patient questionnaire provided 94 self-care practices and free text options in 11 categories: over-the-counter medication, specific foods or drinks, herbal teas, alcoholic drinks, special self-prepared recipes, pastilles or drops, something for the nose, inhalation, throat gargle/spray, items applied externally, and extras at home. In addition, the following patient characteristics were requested: age, gender, health insurance status, number of school years, pills taken daily, and expenditures for the last common cold. To assure random samples at all sites, the questionnaires were distributed to consecutive patients independent of their reason for physician consultation. Inclusion criteria were the following: being ≥18 years, being able to understand the questionnaire, or being in attendance of someone providing assistance.

### 2.3. Patient Involvement

Primary care physicians who developed and conducted the study had a dual role as professionals and as patients who experienced common colds themselves. Results of the COCO study will be disseminated in form of patient information materials.

### 2.4. Sample Size Calculation

To obtain a representative sample for a practice of up to 10,000 patients, a total of 94 patients had to be studied (CI 95%; SE 0.05; accuracy 0.1). This estimate was based on the assumption that the sample of patients surveyed is random for the respective practice. To account for nonresponders, oversampling by 25% was planned, which led to a sample size of 117.5 (94 + 23.5 patients), rounded to 120 patients per primary care setting. This sample size also yielded representative results for items with a prevalence in the range of 40% to 60% in the general population. For details see Weltermann et al. [18].

### 2.5. Statistical Analysis

Self-care items were classified by 2 researchers in terms of their mode of application: “intestinal absorption,” “intranasal application,” “local oral effects,” “inhalation,” “topical use in throat,” “external use,” “foodstuffs,” and “extras at home.” In a subclassification, single items were further subclassified using the ATC (WHO Anatomical Therapeutic Chemical) classification system for pharmacological substances and—if not listed—plausible groups and subcategories. Items were listed individually if they had a utilization rate of at least 1% (*n* ≥ 27) prior to age standardization; otherwise, they were summarized as “other” within each mode.

Descriptive analyses of all self-care practices were performed for the total study population and stratified by country. To account for differences in age distributions between European countries and practice samples, results were age-standardized using the 2013 European Standard Population [19]. Data were analyzed on the basis of five aspects:

1. “Number of self-care practices used”: (a) mean item use; (b) the average number of items per 100 participants stratified by country; and (c) prevalence of using any self-care were considered.
2. “Mode of application”: (a) distribution of all single items reported per mode of application; (b) % of participants using at least one item per mode; and (c) mean item use per mode (subclassification) were considered.
3. “Single items used” (subclassification): (a) the most frequently used single items; (b) the sum of the five most frequently used items per country; (c) frequently used items (≥5% utilization rate); and (d) rarely used items (1% to 5% utilization rate) were considered (selected items used by <1% of the participants were reported).
4. “Evidence-based self-care”: based on 14 Cochrane reviews [15–17, 20–30] and one other review [31], items were grouped according to their evidence of effectiveness for the treatment of common colds in adults. Prevalence of the use of these evidence-based products was expressed as a ratio (non-evidence-based/evidence-based measures). In addition, all items with an ATC code and “antibiotics” were grouped as pharmaceutical products.
5. “Analysis by the countries' purchasing power standard (PPS)”: based on the PPS, countries were classified into three groups. The PPS is an artificial value which is based on each nation's Gross Domestic Product and eliminates price level differences among countries [32]: PPS I included Austria, Sweden, Germany, and Finland; PPS II included France, Israel, Italy, Spain, and Slovenia; PPS III included Poland, Turkey, and Macedonia. Analyses included (a) the mean item use, (b) the use of evidence-based self-care (at least 1, mean), (c) the use of pharmaceutical products (at least 1, mean), and (d) the mean expenditure in the 3 PPS groups.

The analysis included all returned questionnaires. Statistical analyses were performed using IBM® SPSS® Statistics, version 20 and SAS version 9.4 statistical package (SAS Institute, Inc., Cary, NC, USA).

## 3. Results

### 3.1. Characteristics of the Participants

A total of 27 sites from 14 European countries participated in the survey. 3,074 questionnaires were returned. After excluding data from sites with different sampling strategies (patient-physician interviews in Romania; use of an older questionnaire version in Bosnia-Herzegovina; and incomplete questionnaire distribution in 3 of 6 German sites), the total study population consisted of 2,724 participants from 22 sites and 12 countries. See Table 1 for details of the participants and Additional File 1 in Supplementary Material available online at http://dx.doi.org/10.1155/2016/6949202 for characteristics per data sampling site/primary care practice.

62.5% of the participants were female and the mean age was 46.7 years. On average, participants had 12.8 years of formal education (including higher education). 41% of the participants self-reported at least one chronic condition. On average, patients were taking 2 tablets daily and 3.1 (±SD 2.9) tablets in the subgroup of those with at least one daily tablet.

### 3.2. Number of Practices Used

In total, 527 items were reported. Of these, 483 items were reported as free text options. The age-standardized total mean item utilization was 11.5 (±SD 6.0, range: 0–53). In total, 99% of participants used any kind of self-care practices. Comparative analyses for open and closed answers showed mean item utilization of 1.6 (±SD 1.0) and 11.0 (±SD 6.6, range: 0–53), respectively.

Comparisons between countries showed a variation by factor 6.4 in the spectrum size for the stated number of different items per 100 participants (35/100 in Macedonia compared to 224/100 in Turkey). We largely abstained from comparisons between countries, because within-country comparisons showed differences of up to 38% in modes of applications and 4.9% in item utilization (see Additional File 1 in Supplementary Material).

### 3.3. Mode of Application Level

**Figure 1:**
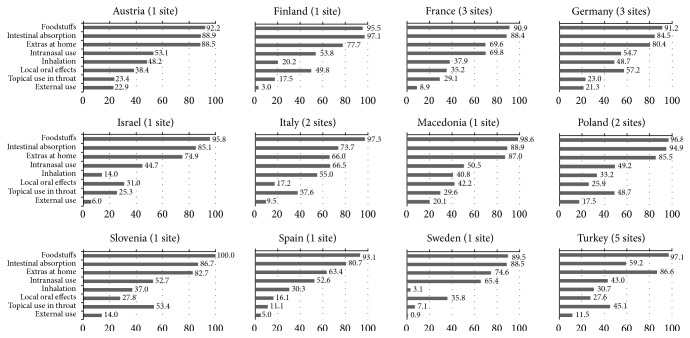
Pattern of self-care by mode of application stratified by country: percentage (%) of using at least 1 item within each mode (weighted by age).

**Figure 2:**
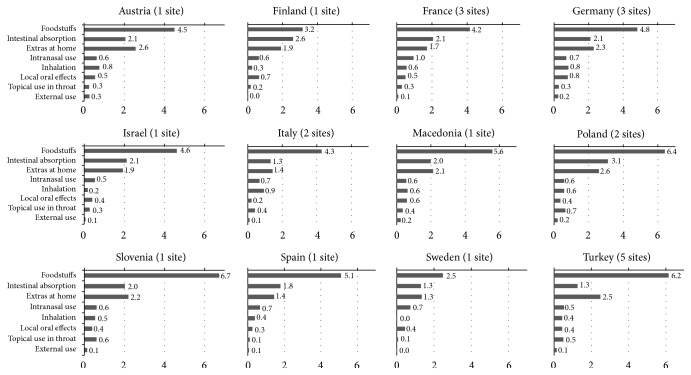
Pattern of self-care by mode of application stratified by country: mean utilization of items within each mode (weighted by age).

Of all 527 items used, “foodstuffs” was reported most frequently (52%). With 276 items, this is also the mode with the largest number of reported items. Much fewer items were reported for “intestinal absorption” (9%, 48 items), “inhalation” (8%, 42 items), “extras at home” (7%, 37 items), “intranasal application” (7%, 36 items), “local oral effects” (6%, 32 items), and “topical use in throat” and “external use” (both 5%, 28 items). Details on the percentages for the utilization of at least one item per mode of application and the mean item utilization for each mode stratified by country are displayed in Figures 1 and 2.

### 3.4. Single Items Used

#### 3.4.1. Self-Care Practices Used by at Least 1% of Participants

After grouping single items based on (a) content (related practices) and (b) utilization rate (≥1%), the spectrum of self-care comprised 97 items. After age-standardization, the most frequently reported single items were water (43%), honey (42%), paracetamol (38%), oranges/orange juice (38%), staying in bed (38%), bath/shower (35%), rest at home (34%), lemons/lemon juice (31%), and chicken soup (30%). Water and/or honey were among the three most frequently used items in 9 countries, oranges/orange juice, staying in bed, and/or pain medication in 5 countries, and rest at home and/or chicken soup in 4 countries. In the total sample, ≥2 of the five most frequent items (water, honey, paracetamol, oranges/orange juice, and staying in bed) were used by 63% of the participants, and 34% used at least 3 of these items.

The five most frequently used self-care practices of each country added up to 19 different self-care practices across all countries: bath/shower, rest at home, staying in bed, chicken soup, lemons/lemon juice, oranges/orange juice, mandarins, other fruits incl. “more fruit,” lime blossom tea, mixed herbal tea, honey, hot milk with honey, water, ibuprofen, paracetamol, vitamin C, nasal decongestants, saline for nose, and pastilles for cough.

An overview on the frequently used self-care practices, that is, items with a utilization rate of at least 5%, is provided in Table 2; items rarely used (1% to 5%) are provided in Table 3. When taking all items into account, fluids of any type were used by 92% of participants, including any tea by 74% and any alcohol-containing liquids by 12%. Fruit and/or juices were used by 70% of participants and vegetables by 24%. Soup was consumed by 31%. With regard to “extras at home,” 56% of participants reported preferring to “stay in bed,” “sleep,” or “rest at home” and 57% used something related to “warmth.” For “intestinal absorption” it is noteworthy that 60% of participants used at least one pain medication. Moreover, although the questionnaire item vitamin C was chosen by 28%, when also considering fruit-based vitamin C (citrus fruits and juices) it is 68%. At least one nose drop/spray was reported by 36% of participants, at least one flavored lozenge by 33%, at least one gargle by 17%, and at least one rub by 10%.

#### 3.4.2. Self-Care Practices Used by Fewer Than 1% of the Participants

Besides the above-reported practices used by at least 1% and 5% of the participants, it is noteworthy to mention some of the 437 practices that are used by less than 1%, some of which can certainly be described as curiosa. Examples are as follows: “topical use in throat”: oil pulling/swishing (*n* = 1), sulfur (*n* = 1), vinegar (and sage) (*n* = 6), soda/baking powder (*n* = 5), and salt and soda (*n* = 4); “external use”: compresses with curd cheese or horseradish (each *n* = 1), rub with olive oil (*n* = 7), and fire cupping (*n* = 13); “inhalation”: cold mist (*n* = 2); “extras at home”: hot light (*n* = 4), physical activity (*n* = 10), and foot bath (*n* = 5); and “foodstuffs”: pickle juice (*n* = 3), amber with alcohol (*n* = 15), Coca Cola (*n* = 3), orchid hot drink (*n* = 2), lemonade (*n* = 1), various mixtures for syrups and syrup-like solid foodstuffs based on honey or sugar in combination with, for example, lemon or ginger (*n* = 21), tripe soup (*n* = 4), or tea with* Inonotus obliquus* (a mushroom), and a shot of wheat (*n* = 1).

### 3.5. Evidence-Based Products Proven to Be Somewhat Effective

In total, 21 self-care items were grouped as evidence-based. See Table 4 for an overview. The mean of evidence-based products was 1.1 items (±SD 0.97) and ranged between 0 and 5 items. Of the 2,691 participants using self-care, solely evidence-based self-care was used by 0.3% of participants, solely non-evidence-based self-care was used by 31.2%, and a combination of both was used by 68.5%. In participants who combine both, participants used 8.0 times more non-evidence-based items than evidence-based items.

An additional 35 items were grouped as non-evidence-based pharmacological products which were used by 4.6% (*n* = 124) of the participants. Any pharmaceutical products—irrespective of their evidence-based status—were used by 62% of the participants. Participants who combined pharmaceutical and nonpharmaceutical products used 9.0 times more nonpharmaceutical products than pharmaceutical products.

### 3.6. Analysis by the Countries' Purchasing Power Standard

The number of items differed between PPS groups: with a mean of 12.4 (±SD 7.2) it was highest in the lowest PPS group (III) and equal—although lower—in PPS II (10.6, ±SD 5.8) and PPS III (10.5, ±SD 6.2). Conversely, the use of evidence-based products decreased with increasing PPS (PPS I: 77%, PPS II: 71%, and PPS III: 58%), as did the use of pharmaceutical products (PPS I: 69%, PPS II: 61%, and PPS III: 54%). The mean items used for evidence-based products and pharmaceutical products differed marginally (with increasing PPS: evidence-based products: 1.2, 1.1, and 0.9; pharmaceutical products: 1.0, 0.8, and 0.8).

An estimate of the expenditure per common cold was reported by 86% (*n* = 1,284) of the participants: 10.8 Euros were spent on average (±SD 13.5; range: 0–250). The amount differed by PPS: PPS I: 13.8, ±SD 16.8; PPS II: 10.4, ±SD 12.1; and PPS III: 8.1, ±SD 9.3.

## 4. Discussion

This is the first study on self-care for common cold with data from several European countries. According to the COCO data, European primary care patients can consistently be considered as high users of self-care for common colds. Prevalence of application modes showed similar patterns across countries, while the number of items on country level varied markedly: the modes “foodstuffs,” “intestinal absorption,” and “extras at home” were the most frequently used modes in 10 countries, with “foodstuffs” representing the most frequently used one across all countries. Of the large spectrum of 527 practices reported, 19 self-care practices rank among the top five across countries.

### 4.1. Strengths and Limitations of the Study

This is the first study to analyze prevalence and patterns of self-care for common colds on a larger European scale. Contrary to previous studies it used a rather explorative approach and did not restrict self-care to the use of OTC or home remedies. Age standardization was used to account for differences in ages between sites and countries. This study was not without limitations. Firstly, due to the sampling strategy, results are representative only for primary care practices/sites. Therefore, we largely abstained from country comparisons. Secondly, we did not ask for the participants' reason for using certain self-care practices (e.g., reduction of duration, symptomatic relief, and targeted symptoms) and how they appraise the evidence. Thirdly, we did not ask whether patients' current physician consultation was due to an acute cold. Fourthly, coding unspecific answers might have led to an underrepresentation of pharmaceutical products. Women were overrepresented in our sample, likely due to generally more physician visits by women and the feminization of the elderly population. In addition, participants with more school years are overrepresented as compared to the EU population.

### 4.2. Comparison with Other Studies

The observed prevalence of self-care utilization close to 100% is higher than reported in other studies. For instance, the prevalence was only 84% in the British general population [14] and 69% in Estonian pharmacy customers [33]. In addition, we showed that patients engage in a variety of self-care practices, as the spectrum of reported self-care items among the sites in the 12 countries comprised more than 500 items, on average 12 items per person. Few studies are available for comparison. Irrespective of the health issue, German primary care patients (*n* = 480) used on average 22 home remedies [9]. Specifically regarding colds, the Estonian survey showed average utilization of 4.1 medicinal plants alone [33], and in an American survey among parents (*n* = 153) with a focus on children 3.8 (European Americans) and 3.2 (African Americans) items were reported on average [34].

Turning to self-medication, 62% used pharmaceutical products, which is higher than the 52% reported in the British survey (*n* = 4,327) [13] and the 44% reported in Estonian pharmacy customers (*n* = 300) who used a combination of herbal products/medicinal plants and self-medication [33]. Based on a review of the literature, we identified 21 self-care items in the COCO data that are discussed as possibly being beneficial for cold symptoms in adults; the majority of items lack an assessment for effectiveness or were shown to be ineffective. Of the five most frequently reported items (utilization of about 40% each), that is, “water,” “honey,” “paracetamol,” “oranges/orange juice,” and “staying in bed,” only paracetamol is evidence-based. Overall with two thirds, the majority of participants engaged in self-care that includes both evidence-based items and items without proof of evidence.

The most frequently reported evidence-based items were nasal decongestants followed by the various pain medications (NSAIDs and paracetamol). Examples for non-evidence-based self-care are that one third of respondents still use vitamin C and another 15% use garlic, which can both be considered treatment myths [22, 26]. Similarly, the use of inhalations by about one third is not supported by evidence [28]; the same applies for chest rubs [31]. With 12%, also the non-evidence-based use of alcohol—either drunk alone or added to other liquids—is surprisingly high.

## 5. Conclusions

This study adds to the knowledge on the prevalence and patterns of cold self-care and clearly demonstrates that self-care for cold is frequent, even among those with relatively low utilization. As such, the data are representative on the level of the participating primary care practices. Overall, the documented commitment for self-care is in line with European wide attempts to promote self-care for minor illnesses in order to reduce health care system expenditures and the workload of general practitioners. Nevertheless, given the poor evidence base, the high engagement in self-care highlights the need for patient-directed information. Such information should address the evidence base and safety of self-care practices. Furthermore, studies that evaluate the effectiveness of the frequently used, poorly studied items for common colds are needed.

## Supplementary Material

Descriptives of countries with more than 1 site regarding participants' age, gender, years of school, item utilization and use of at least 1 item within each mode, weighted by age using the European Standard Population 2013 (n = 2,724).

## Figures and Tables

**Table 1:** Characteristics of participants (*n* = 2,724).

	*n*	%
Gender, female (*n* = 2,654)	1,659	62.5%

Insurance status, public (*n* = 2,587)	2,494	96.4

Nonsmoker (*n* = 2,638)	2,052	77.8

Patients with ≥1 self-reported chronic condition	1,086	39.9
Hypertension	608	56
Chronic pain/arthritis	257	23.7
Heart disease	242	22.3
Diabetes	221	20.3
Asthma/chronic bronchitis	197	18.1
Depression	143	13.2
Chronic kidney disease	55	5.1

Age, mean ± SD (*n* = 2,644)	46.7 ± 16.8	

Years of formal education, mean ± SD (*n* = 2,557)	12.8 ± 4.49	

Number of tablets used daily, mean ± SD (*n* = 2,318)	2.0 ± 2.7	
Patients with ≥1 tablet daily (*n* = 1,467)		
ASA/aspirin	314	21.4
Oral contraceptive	165	11.2
Anticoagulants	80	5.5

Participating countries (*n* = 2,724)		
Austria°	110	4
Finland^**#**^	107	3.9
France, 3 sites^+#^	325	11.9
Germany, 3 sites^+°#^	385	14.1
Israel^+^	123	4.5
Italy, 2 sites^#°^	161	5.9
Macedonia°	364	13.4
Poland, 2 sites^+^	241	8.8
Slovenia°	119	4.4
Spain^+^	86	3.2
Sweden^+^	98	3.6
Turkey, 5 sites^°+^	605	22.2

°Mixed (urban/rural); ^+^urban; ^#^rural.

**Table 2:** Frequently used (≥5%) self-care practices on item level (*n* = 2,724), weighted by age^*∗*^.

	*n*	%
*Foodstuffs (95.2% ≥ 1 item; mean use: 5.2)*		

*Tea*		
Fruit		
Lemon + honey	495	**18.0**
Cranberry	181	**6.6**
Herbal		
Peppermint/mint	474	**17.9**
Chamomile	477	**17.3**
Sage	442	**16.8**
Lime blossom	371	**14.0**
Ginger	349	**13.4**
Thyme	208	**7.9**
Cinnamon	202	**7.9**
Mixed	468	**17.7**
Black/green	299	**11.0**
Other	161	**6.3**

*Fruits*		
Oranges/orange juice	1,029	**38.1**
Lemons/lemon juice	837	**31.1**
Mandarins	684	**25.2**
Grapefruits/grapefruit juice	185	**6.7**
Other fruits incl. “more fruit”	798	**29.4**

*Vegetables*		
Garlic	423	**15.2**
Other vegetables incl. “more vegetables”	396	**14.0**

*Honey*		
Honey	1,138	**41.9**
Hot milk + honey	531	**19.3**

*Alcohol*		
Tea with rum	137	**5.0**

Water	1,168	**42.8**
Chicken soup	811	**29.8**

*Intestinal absorption (80.8% ≥ 1 item; mean use: 1.9)*		

*Pain medication*		
Paracetamol	1,019	**38.2**
Ibuprofen	417	**15.7**
ASA/aspirin	384	**13.7**
“Pain medication” (unspecific)	319	**12.0**

*Cough medicine*		
Syrup for cough	516	**18.5**
Pastilles for cough	419	**15.4**
Drops for cough	228	**8.2**

Vitamin C	758	**28.2**
“Pills for cold” (unspecific)	528	**19.9**
“Homeopathics” (unspecific)	183	**6.9**

*Extras at home (80.5% ≥ 1 item; mean use: 2.1)*		

Staying in bed	1,020	**38.1**
Bath/shower	930	**35.3**
Rest at home	909	**33.7**
Warm clothes	735	**27.2**
Open windows	585	**22.0**
Warm covers	495	**18.6**
Take a day/days off	370	**14.4**
Hot water bottle	303	**11.3**
Pray for recovery	224	**8.5**

*Intranasal application (53.1% ≥ 1 item; mean use: 0.7)*		

Decongestants	703	**26.5**
Saline for nose	479	**18.1**
Nose drops/spray (unspecific)	287	**10.8**
Nasal washings	216	**8.2**

*Inhalation (36% ≥ 1 item; mean use: 0.6)*		

Peppermint/menthol	399	**14.6**
Saline	182	**7.0**
Chamomile	142	**5.1**
Pot with steaming water (unspecific)	390	**14.3**

*Local oral effects (35.3% ≥ 1 item; mean use: 0.5)*		

*Lozenges*		
Lemon	296	**10.7**
Eucalyptus	269	**10.0**
Sage	260	**9.6**
Peppermint	241	**9.1**

*Topical use in throat (33.2% ≥ 1 item; mean use: 0.4)*		

*Gargle*		
Saline	283	**10.2**
Sage	187	**6.8**

*Throat spray*	401	**15.2**

*External use (13.6% ≥ 1 item; mean use: 0.2)*		

Rub with essential oils	172	**6.5**

^*∗*^European Standard Population 2013.

**Table 3:** Rarely used (1% to <5%) self-care practices on item level (*n* = 2,724), weighted by age^*∗*^.

	*n*	%
*Foodstuffs*		

*Tea*		
Fruit		
Elderberry	124	**4.7**
Raspberry	109	**3.9**
Apple peel	62	**2.4**
Other/unspecific	555	**2.3**
Herbal		
Marshmallow root	129	**4.8**
*Echinacea* propolis	104	**4.0**
*Eucalyptus*	91	**3.3**
Pine syrup	73	**2.7**
St. John's wort	70	**2.6**
*Verbena*	37	**1.4**
Other/unspecific	73	**2.7**

*Alcohol*		
Mulled wine	104	**3.8**
Alcohol added to other liquids	39	**1.4**
Beer/wine	36	**1.2**

Onion cough syrup	112	**4.1**
Milk with butter	106	**3.9**
Onion juice with honey	100	**3.8**
Food with marjoram	86	**2.9**
Other solid food	66	**2.5**
Soup: other/unspecific	49	**1.9**
Other liquids	45	**1.7**

*Intestinal absorption*		

Calcium	126	**4.3**
Zinc	64	**2.4**
Plantago cough syrup	65	**2.3**
Umckaloabo	35	**1.4**

*Extras at home*		

Electric warming blanket	55	**2.1**

*Inhalation*		

Eucalyptus (oil)	129	**4.8**
Essential oil (unspecific)	117	**4.5**
Camphor (oil)	27	**1.0**

*Local oral effects*		

Herbal lozenges (unspecific)	127	**4.8**
Cinnamon lozenges	37	**1.4**
Oral antiseptics	35	**1.4**

*Topical use in throat*		

Gargle with chamomile	71	**2.5**
Anti-infectives	44	**1.6**

*External use*		

Chest rub with alcohol	93	**3.5**
Calf compress	61	**2.3**
Cupping	54	**2.0**

^*∗*^European Standard Population 2013.

**Table 4:** Use of evidence-based self-care (*n* = 2,724), weighted by age^*∗*^.

	*n*	%
Paracetamol (N02BE01)^*∗∗*^	1008	37.8
Nasal decongestant, unspecific	687	25.9
Ibuprofen (M01AE14)	417	15.8
Acetylsalicylic acid (ASA) (N02BA01)	384	13.7
Pain medication, unspecific	319	12
*Pelargonium sidoides* (umckaloabo)	35	1.4
Paracetamol in combinations (N02BE51)	16	0.6
Mefenamic acid (M01AG01)	4	0.2
Metamizole (N02BB02)	6	0.2
Xylometazoline (R01AA07)	4	0.2
Naphazoline (R01AA08 )	2	0.1
Pseudoephedrine (R01BA02)	2	0.1
Diclofenac (M01AB05)	1	0.0
Naproxen (M01AE02)	1	0.0
Other antihistamines for systemic use (R06AX)	1	0.0
Loratadine (R06AX13)	1	0.0
Fexofenadine (R06AX26)	1	0.0
Ephedrine (R01AA03)	1	0.0
Oxymetazoline (R01AA05)	1	0.0
Sympathomimetics, combinations excl. corticosteroids (R01AB)	1	0.0
Pseudoephedrine, combinations (R01BA52)	1	0.0

^*∗*^European Standard Population 2013; ^*∗∗*^in parenthesis: ATC code if available.

## Abbreviations

ATC code: WHO (World Health Organization) Anatomical Therapeutic Chemical code
CI: Confidence interval
COCO: Common Cold Study
EGPRN: European General Practice Research Network
ESP: European Standard Population
EU: European Union
NSAID: Nonsteroidal Anti-inflammatory Drugs
OTC: Over-the-counter medications
PPS: Purchasing power standard
SD: Standard deviation
WHO: World Health Organization.

## Additional Points

*Trial Status.* Participants' recruitment was finished in April 2014.
