# Supplementary material for: Self-Care for Common Colds by Primary Care Patients: A European Multicenter Survey on the Prevalence and Patterns of Practices—The COCO Study
Source: Evid Based Complement Alternat Med. 2016 Sep 21;2016:6949202. doi: 10.1155/2016/6949202 (PMC5050368; doi:10.1155/2016/6949202)
Supplement: Supplementary file 1 — Descriptives of countries with more than 1 site regarding participants' age, gender, years of school, item utilization and use of at least 1 item within each mode, weighted by age using the European Standard Population 2013 (n = 2,724). [file 6949202.f1.docx]

Additional File 1 Descriptives of countries with >1 site for age, gender, years of school, item utilization and use of at least 1 item within each mode, weighted by age* (n=2,724).

|  | **Site no.** | **Total n** | **Age, mean** | **Female, %** | **Years of school, mean** | **Urban/ rural/ mixed** | **Item use, mean** | **Food-stuffs** | **Intesti-nal absorp.** | **Extras at home** | **Intrana- sal use** | **Inhala- tion** | **Local oral effects** | **Topical use in throat** | **Exter-nal use** |
| --- | --- | --- | --- | --- | --- | --- | --- | --- | --- | --- | --- | --- | --- | --- | --- |
|  |  |  |  |  |  |  |  |  |  |  |  |  |  |  |  |
|  |  |  |  |  |  |  |  |  |  |  |  |  |  |  |  |
| **Germany** | 1 | 101 | 49 | 52.8 | 11.8 | urban | 12.2 | 92.6 | 82.6 | 78.4 | 56.5 | 38.0 | 56.8 | 18.7 | 17.9 |
|  | 2 | 125 | 57 | 67.5 | 11.2 | mixed | 10.5 | 85.5 | 80.7 | 74.1 | 47.0 | 36.7 | 56.0 | 19.4 | 20.3 |
|  | 3 | 159 | 45.4 | 69.9 | 12.6 | rural | 13.1 | 94.3 | 88.2 | 85.9 | 58.7 | 63.0 | 58.2 | 28.0 | 24.1 |
| **Poland** | 1 | 120 | 41.6 | 58.3 | 15.1 | urban | 14.1 | 96.5 | 93.3 | 85.2 | 49.8 | 28.4 | 20.0 | 49.1 | 16.8 |
|  | 2 | 121 | 45.8 | 70.2 | 15.2 | urban | 15.0 | 97.1 | 96.7 | 85.8 | 48.5 | 38.6 | 32.5 | 48.3 | 18.3 |
| **Turkey** | 1 | 120 | 44.6 | 54.2 | 8.9 | urban | 9.1 | 95.2 | 36.9 | 80.6 | 28.8 | 45.1 | 18.0 | 39.0 | 2.7 |
|  | 2 | 120 | 42.0 | 50.8 | 11.3 | mixed | 14.0 | 97.4 | 59.7 | 91.0 | 56.1 | 25.1 | 30.2 | 45.0 | 12.0 |
|  | 3 | 120 | 39.3 | 68.9 | 14.4 | urban | 13.9 | 97.4 | 74.6 | 87.6 | 50.5 | 32.1 | 36.4 | 50.8 | 8.8 |
|  | 4 | 120 | 40.6 | 65.3 | 11.5 | mixed | 13.1 | 98.3 | 69.5 | 91.4 | 44.2 | 27.6 | 23.8 | 51.6 | 10.1 |
|  | 5 | 125 | 45.4 | 62.4 | 7.9 | - | 9.7 | 97.5 | 55.7 | 82.6 | 35.6 | 23.7 | 29.6 | 39.2 | 23.8 |
| **France** | 1 | 109 | 53.4 | 66.1 | 16.6 | urban | 8.9 | 91.6 | 81.9 | 72.0 | 73.7 | 32.9 | 24.6 | 19.6 | 6.2 |
|  | 2 | 107 | 39.1 | 64.5 | 14 | urban | 11.2 | 93.2 | 93.0 | 69.4 | 56.3 | 41.0 | 35.8 | 28.8 | 8.7 |
|  | 3 | 109 | 44.1 | 63.3 | 15.4 | rural | 10.8 | 88.0 | 89.7 | 67.7 | 79.5 | 39.5 | 44.3 | 38.1 | 11.4 |
| **Italy** | 1 | 121 | 61.2 | 56.2 | 11.6 | mixed | 9.3 | 98.2 | 71.0 | 69.3 | 67.3 | 52.9 | 15.7 | 30.9 | 9.9 |
|  | 2 | 40 | 54.5 | 55 | 12.5 | rural | 9.6 | 94.8 | 81.4 | 56.4 | 64.1 | 61.0 | 21.5 | 56.8 | 8.1 |

*European Standard Population 2013
